# Alejandro Daly: bridging the gap between climate and health

**DOI:** 10.2471/BLT.26.030326

**Published:** 2026-03-01

## Abstract

Alejandro Daly speaks with Gary Humphreys about raising awareness of air quality, engaging young people in the climate and health conversation, and addressing the ethical dimensions of climate research.

*Q: What inspired your journey into climate and health advocacy?*

A: I was born with asthma, so from a young age I really struggled with the polluted air in Valencia, [Bolivarian Republic of] Venezuela, the city where I grew up. Then in 2014, when I was 17, there was some political upheaval in my country and my siblings and I had to move to Bogotá in Colombia. The air pollution was even worse there. It really affected me, and I saw a lot of people around me struggling too. In 2018, there was a bad pollution event, and I started thinking something had to change. That's when I got involved with an advocacy group called *El derecho a no obedecer* (The right to not obey), which focused on empowering young people to become active participants in shaping their communities and holding institutions accountable. We wanted to raise public awareness about the air quality problem and put pressure on the government to act. We’d heard about a group in Medellín, Colombia, that had put giant black face masks on the Botero sculptures in the city to protest the poor air quality, and we thought that was really cool. Then, one day, during a brainstorming session, someone said, “I wish people could see what air pollution is doing to our health, like, actually see it.” And that’s when it clicked. We were like, “yes, exactly. Let’s find a way to literally show people what it looks like”.

*Q: What did you do?*

A: We made a giant pair of lungs out of white cotton. The model was about two meters tall. We set it up in one of Bogotá’s busiest public transport stations, and over the next few months documented how it went from being white to being almost black because of the pollution. It really got people’s attention, including this policeman who came up to us one day and asked us what we thought we were doing. Now, this was not great because we did not actually have a permit for the thing. But when I told him what it was and what it was trying to communicate, he got a look on his face. “Oh wow,” he said, “do you think my lungs look like that? This is my station. I work here every day.” I could see that it really hit home with him. That was a turning point for me. It made me realize the power of using visual symbols to help people understand that the environmental and climate crisis is also a public health crisis. And, by the way, he let us keep the lungs there for a long time despite the fact that we did not have a permit. 

*Q: You are best known for co-founding the Latin American Coalition for Clean Air. How did that come about?*

A: The idea really came out of my work with *El Derecho a no obedecer*, which I started directing in 2019. We were running campaigns, hosting dialogues and using creative approaches to tackle big social issues, things like civic engagement, public health and environmental justice. We connected with young people across Colombia and other parts of Latin America, and one issue kept coming up: air pollution. It was something that touched all of us, but it was not getting nearly enough attention and there were no strong networks in the region focused on clean air as a human right. That’s what pushed me to help launch the Latin American Coalition for Clean Air. It was a space where youth activists, researchers and organizations could come together to fight for clean, breathable air and fair environmental policies. Our goal has always been to put clean air on the public and political agenda, and to highlight how environmental harm ties directly into health and inequality. Since then, we’ve run awareness campaigns, trained thousands of young leaders and worked hand-in-hand with communities to monitor air quality and push for real change.

“There needs to be more research in the countries most impacted.”

*Q: You’ve made a point of engaging children in air quality data collection. What was the motivation behind that?*

A: From the very beginning, we believed that young people had the capacity to lead the fight for clean air and that monitoring air quality was a crucial part of that. We started a schools-based initiative with partners where we trained kids to use low-cost air quality sensors in their neighborhoods. For many of them, it was the first time they had done anything like scientific research, and they were incredibly impressive, very curious and determined. Over the years, we’ve trained over 600 students in public schools, and now there are more than 60 sensors spread across the country, generating real data from the ground.

*Q: Has any of that data been used in academic research?*

A: Only once, formally. We have good relationships with the scientific community. We invite researchers to speak at our events and engage with our young people, but because of variability in how the kids collect data, it’s not always seen as rigorous enough for academic studies. Still, that doesn’t mean the data are not valuable. They have been used to drive community conversations and pressure local governments. The kids use them to hold leaders accountable, to learn and to raise awareness. We also run workshops in schools and community centres where participants learn about the sources of pollution and its health effects. Honestly, watching children step into roles as researchers and advocates has been one of the most inspiring parts of my work. Some of them have even taken their voices to the global stage. I have had the privilege of seeing them speak at international events, representing their communities and demanding that world leaders take youth and environmental justice seriously. I was at a public health event in Costa Rica in February 2025, and two kids I had trained in Bogotá were there, now leading advocacy efforts in their own public school. They don’t just share statistics, they share their reality. 

*Q: To what extent does that make a difference?*

A: Well, given the slow progress we’re making towards our stated climate and environmental goals, you have to wonder, but I am convinced that making sure young people are heard is key. That’s why I think we need to do more to uplift young voices. We all know names like Greta Thunberg or Vanessa Nakate, and they’ve done incredible work, but air pollution still does not get the space it deserves in climate conversations, especially in terms of its links with health. I’ve spoken at a lot of events, including multiple Conferences of the Parties, and often I’m the only one talking about air pollution. And the link between climate and health is only just starting to break through, partly thanks to the World Health Organization. But more organizations need to step up, especially when it comes to supporting youth from the Global South. Not just including them as a token gesture, but really investing in them, elevating their voices, and recognizing them as experts. This is true of Global South researchers generally, of course.

“There are things I believe climate researchers […] should never do.”

*Q: Can you say more about that?*

A: There needs to be more research in the countries most affected. There isn’t nearly enough, and it’s deeply frustrating. The way research is financed and supported globally today often promotes voices from the Global North. While, people who are actually living in and working on the frontlines of air pollution, whether in Colombia, Mongolia or elsewhere, are left out. Similarly, too often, influencers are given the microphone simply because they post about climate change on social media. Which begs the questions, “are you an advocate just because you post about climate on social media? Or are you an advocate because you are changing something on the ground?” For me, it’s the latter.

*Q: Where do you stand on the question of community engagement in research?*

A: I have always tried to promote community-first research and advocacy, where the people affected are also those who shape our understanding of the challenges and the solutions needed to meet them. There is an important ethical dimension here. I’ve always come to this from the advocacy side, so my ethics around this have been very clear from the beginning. There are things I believe climate researchers and advocates should never do, like enabling deforestation or, for example, delaying decarbonization in order to support research projects. Doing so makes research a kind of extractive industry, where information is gathered despite the harms done to local communities and without ever allowing those communities to benefit from the information generated. Too often, data are taken from communities, published in journals and never returned to the people who provided it.

*Q: What are you currently working on?*

A: I’m doing a lot of advocacy through the Latin American Coalition for Clean Air, making sure that grassroots air pollution advocates have a strong presence. We sent a delegation to the United Nations climate change conference, which took place in November 2025 in Belém, the capital of Pará state in Brazil. This was the first time a Conference of the Parties was held in the Amazon region. A lot of the air pollution in our region, including in [Bolivarian Republic of] Venezuela, Brazil, Colombia and Ecuador, comes from Amazon forest burning, so the conference was a unique opportunity to talk about that and to make the connection between climate, health and deforestation. My other main focus is philanthropy, trying to mobilize funds for the cause. And personally, I’m still figuring out how to inspire more young people to speak out about air pollution and shape climate narratives with a health lens. It’s still an open question, how to do that effectively. I don’t have the full answer yet, but I’m working on it.

